# Upper Extremity Arteriovenous Malformation Masquerading as a Biceps Ganglion Cyst

**DOI:** 10.7759/cureus.38349

**Published:** 2023-04-30

**Authors:** Anderson Lee, Paige Chapman, Kiran Boyinepally, Abdul A Mustapha

**Affiliations:** 1 Department of Orthopaedic Surgery, The University of Toledo Medical Center, Toledo, USA

**Keywords:** vascular, upper extremity, diagnostic, arteriovenous malformation, ganglion cysts

## Abstract

A 71-year-old male presented to the clinic with two multiloculated cystic masses located over the distal bicep of his left upper extremity, causing discomfort when flexing the extremity. Referral and point-of-care ultrasound at an outside location suggested cystic masses that were consistent with that of a ganglion cyst. Physical exam and clinical presentation were also consistent with this diagnosis. Due to the patient’s symptomatic presentation, surgical intervention was proposed, and the patient consented. Intraoperatively, it was discovered that the patient’s mass was unexpectedly due to an arteriovenous malformation, revealing the importance of careful workup for cystic masses presenting in unusual locations.

## Introduction

Ganglion cysts are common, mucin-filled cysts that typically occur on the hands, especially the dorsal aspect of the wrist. Oftentimes, they are asymptomatic but may cause discomfort due to enlargement or movement that can cause irritation over the area [1]. There have been reported incidents of ganglion cysts growing in unusual locations such as the long head of the biceps tendon, which is proximal to the intertubercular groove [2,3]. Symptomatic ganglion cysts can be treated with surgical excision, a safe and relatively simple procedure that poses minimal risk [3]. We present a case in which a 71-year-old male who was diagnosed with a ganglion cyst based on ultrasound and clinical presentation was found to have a clinically symptomatic arteriovenous malformation over the left upper extremity antecubital fossa. Informed consent to present this case was obtained directly from the patient.

## Case presentation

**Figure 1:**
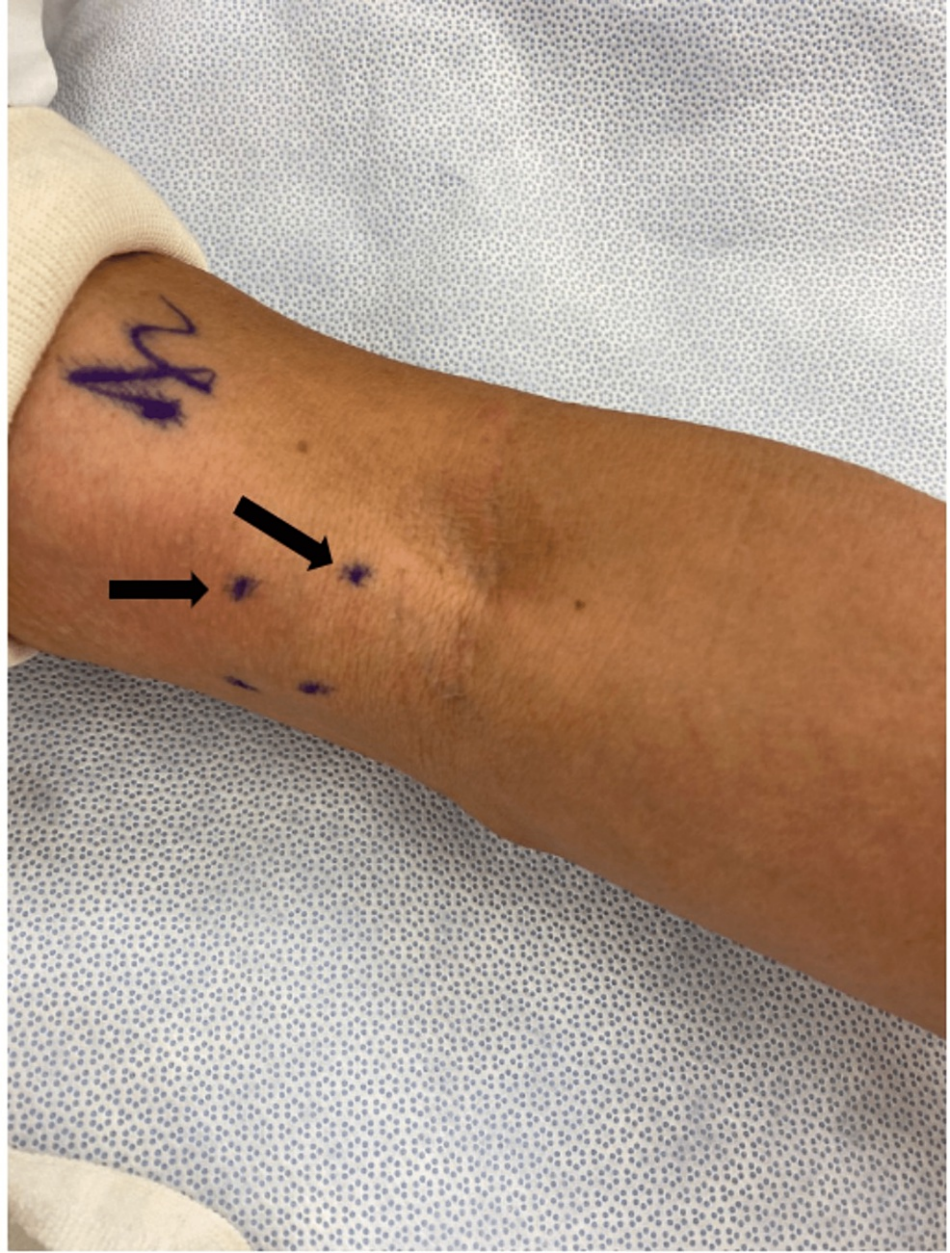
Two multiloculated cystic masses over the medial aspect of the distal bicep immediately proximal to the elbow flexion crease (black arrows)

**Figure 2:**
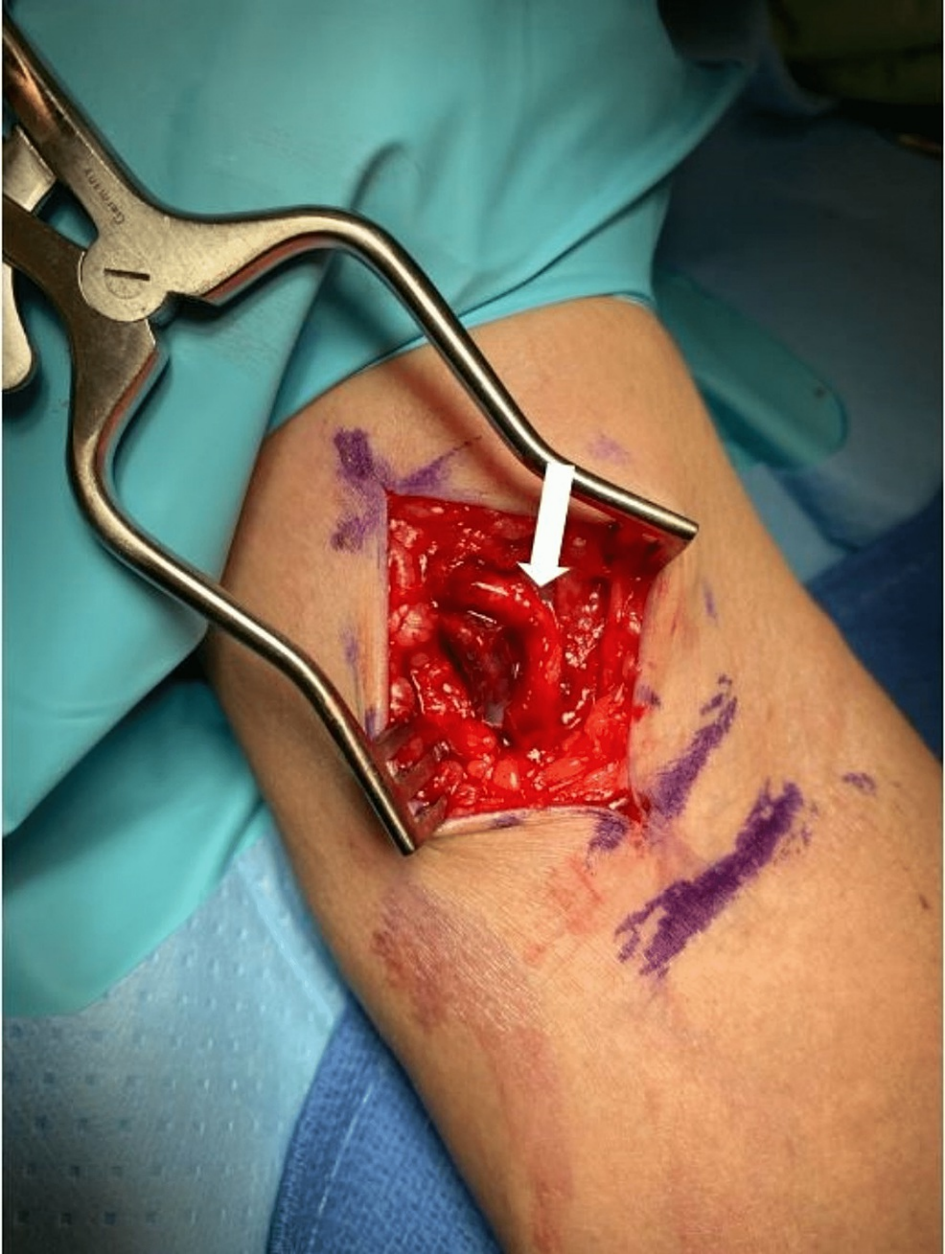
Intraoperative image of an arteriovenous malformation over the distal bicep of the left upper extremity (white arrow)

This is a case of a 71-year-old male who presented to our clinic for evaluation of a left antecubital fossa mass. The patient had previously been seen by internal medicine at another location in which a point-of-care ultrasound had been performed, revealing a fluid-filled mass. No Doppler ultrasound findings had been sent to us. He stated that the mass had been developing over multiple years until a point at which he experienced discomfort at the site of the mass when flexing his elbow greater than 90 degrees and decided to receive an evaluation. The patient had no known or significant prior surgical or medical history. Physical examination of the left upper extremity revealed an approximately 2 x 1.5 cm palpable multiloculated cystic mass over the medial aspect of the distal bicep just proximal to the elbow flexion crease (Figure 1). The mass was nontender to palpation and the patient only expressed discomfort when flexing the elbow. No pulsatile structures were palpable and there were no color changes to the skin. The consistency of the mass was fluid-like and resembled that of a ganglion cyst, and no other abnormal physical findings were found. Due to the patient’s symptomatic cyst, surgical exploration of the mass was offered to the patient to which he consented following a discussion of the risks and benefits. MRI was suggested, but it was felt that this would further delay the surgery and the patient already had an ultrasound read that showed a cystic structure overlying the distal biceps tendon. The patient was taken back to the operating room 25 days later. Anesthesia was smoothly induced, a tourniquet was applied to his left upper extremity, and the patient was draped in the standard sterile fashion. Sharp and blunt dissection was carried out after an appropriate surgical timeout with special attention so as to not injure any neurovascular structures. As dissection continued, it was revealed that the mass was not actually a ganglion cyst but rather an arteriovenous malformation (AVM) (Figure 2). Given that the mass was not a ganglion cyst, it was elected to leave the malformation as found and the wound was closed. At the one-week follow-up postoperatively, the patient denied any symptoms of numbness, tingling, or paresthesias and had no issues with the incision site. The patient was advised that if the AVM continued to bother him to follow up with vascular surgery.

## Discussion

Ganglion cysts are mucin-filled cysts that contain paucicellular connective tissue, with 70% of cases located on the dorsal aspect of the wrist [1]. Most cases are asymptomatic, though many patients may complain of the appearance, tenderness, or weakness worsened with wrist motion. Diagnostic imaging is typically completed with radiographs to rule out bone manifestations or with MRI if a tumor is suspected. Ultrasounds may also be performed to avoid damage to the radial artery when performing a needle aspiration [1]. Asymptomatic cases may be observed for spontaneous resolution, but a high rate of recurrence typically results in surgical excision. Ganglion cysts located on the biceps are a rare occurrence that has been reported a handful of times in literature. Most commonly, they occur on the long head of the biceps tendon as it traverses the glenohumeral joint [2]. Other incidences have been reported in which a ganglion cyst was located on the biceps tendon in the intertubercular groove [3]. Due to the location of these cysts and the surrounding structures of the shoulder capsule, magnetic resonance imaging (MRI) and Doppler ultrasound can be performed to better visualize the mass and confirm the diagnosis. Confirmation of the diagnosis is typically consistent with a lobulated collection on MRI or a negative signal and a hypoechoic mass on Doppler imaging [2]. Among these rare occurrences of ganglion cysts, one report highlighted a unique possibility of proximal median nerve entrapment by the biceps tendon cyst with immediate resolution following ultrasound imaging, cyst aspiration, and a corticosteroid regiment [4]. Thus, it may be extremely beneficial to monitor the growth of the multiloculated cysts by following a similar protocol with diagnostic imaging followed by surgical exploration. In this patient’s case, his relatively unremarkable surgical and medical history, ultrasound results, and initial clinical presentation were consistent with a uniquely located ganglion cyst. However, surgical exploration revealed an AVM rather than a ganglion cyst, which required vascular consultation as opposed to orthopedic management.

Arteriovenous malformations are abnormal connections between arteries and veins, often found in the brain with symptoms of headache and seizure. They pose a high risk of rupture and hemorrhage, with computed tomography angiography (CTA) revealing feeding vessels that can emanate from major arteries and veins. Patients with AVMs pose a high risk of life-threatening hemorrhage and are treated with pre-emptive surgical ligation [5]. Upper extremity AVMs outside the brain constitute only 10% of the total AVMs and among those, a majority of the cases present in the hands [6]. Thus, instances of arteriovenous malformations masquerading as cystic structures can occur as uncommon presentations that can lead to misdiagnosis if a Doppler examination is not routinely performed to assess flow characteristics. In one case, a 26-year-old male with left flank fullness and mild on-and-off hematuria underwent a routine external ultrasound of the abdomen and was subsequently diagnosed with a simple renal cyst. Further evaluation with a contrast-filled CT scan ended up revealing the final diagnosis of an arteriovenous (AV) fistula of the renal artery and vein with an aneurysm [7]. We highlight this case as an example of how an uncommon clinical presentation and atypical ultrasound protocol may lead to the misdiagnosis of an AVM masquerading as a cyst.

In the realm of orthopedics, ganglion cysts are a common and uncomplicated problem that usually does not require extensive diagnostic imaging or workup. In atypical situations such as this one, where a cyst-like mass was found on the distal biceps, further workup should be performed to rule out other abnormalities. While point-of-care ultrasound was performed, revealing a cystic mass, it is advantageous to perform further workup with Doppler ultrasound or MRI, as this case reveals how an arteriovenous malformation can masquerade as a ganglion cyst.

Even though no complications arose from surgery, utilization of the aforementioned imaging options may have prevented the decision to pursue a relatively invasive approach and monitor the blood vessels instead. From the vantage point of vascular surgery, the progression of AVM symptomatology can be tracked via Schobinger’s four-stage scale to help inform further ameliorative or surgical options (Table 1) [8].

**Table 1:** Schobinger's four-stage scale for evaluation of arteriovenous malformation symptomatology

Stage	Features
I (Quiescence – Resting Stage)	Warm, pink-blue appearance, and shunting evident from Doppler scan
II (Expansion)	Enlargement, pulsation, tortuosity of veins, audible bruit
III (Destructive)	Dystrophic (disruptive) skin changes, painful bleeding, and ulceration
IV (Decompensation)	Cardiac Failure 2º to volume overload and congestion

One case illustrated in the literature described a woman who presented with a bilobed palpable lesion over her left antecubital fossa. This was eventually diagnosed as a brachial artery aneurysm by means of a contrasted-enhanced MRI, proximal to a previous AVM arising from the dorsal interosseous and ulnar arteries that had been treated before by embolization. In this case, her presentation of an AVM and aneurysm of the peripheral vasculature was quite rare. However, the palpable mass was due to her aneurysm rather than the AVM itself [9].

## Conclusions

Although ganglion cysts and arteriovenous malformations are common disease processes, cystic masses that are present in the antecubital fossa are a rare and unique occurrence. If there is a concern for a cystic mass in an unusual anatomic location, there should be further preoperative workup and advanced imaging for this presentation. Ultrasounds should be performed with Doppler studies of cystic masses to rule out vascular cases. Further imaging with MRI may also be performed to better visualize structures and properly diagnose issues. In this case, while a prior ultrasound read demonstrated a fluid-filled mass, extra precaution should be taken in order to better visualize and properly identify the pathology.
